# Trophoblast Cell Function in the Antiphospholipid Syndrome

**DOI:** 10.3390/biomedicines11102681

**Published:** 2023-09-30

**Authors:** Svetlana Vrzić Petronijević, Aleksandra Vilotić, Žanka Bojić-Trbojević, Sanja Kostić, Miloš Petronijević, Ljiljana Vićovac, Milica Jovanović Krivokuća

**Affiliations:** 1 University of Belgrade, Faculty of Medicine, University Clinical Center of Serbia Clinic for Obstetrics and Gynecology, Koste Todorovića 26, 11000 Belgrade, Serbia; 2 University of Belgrade, Institute for the Application of Nuclear Energy, Department for Biology of Reproduction, Banatska 31b, 11080 Belgrade, Serbia

**Keywords:** antiphospholipid syndrome, antiphospholipid antibodies, placenta, trophoblast, ncRNA

## Abstract

Antiphospholipid syndrome (APS) is a complex thrombo-inflammatory autoimmune disease characterized by the presence of antiphospholipid antibodies (aPL). Women with APS are at high risk of recurrent early pregnancy loss as well as late obstetrical complications—premature birth due to placental insufficiency or severe preeclampsia. Accumulating evidence implies that vascular thrombosis is not the only pathogenic mechanism in obstetric APS, and that the direct negative effect of aPL on the placental cells, trophoblast, plays a major role. In this review, we summarize the current findings regarding the potential mechanisms involved in aPL-induced trophoblast dysfunction. Introduction on the APS and aPL is followed by an overview of the effects of aPL on trophoblast—survival, cell function and aPL internalization. Finally, the implication of several non-coding RNAs in pathogenesis of obstetric APS is discussed, with special emphasis of their possible role in trophoblast dysfunction and the associated mechanisms.

## 1. Introduction

Antiphospholipid syndrome (APS) is a systemic autoimmune disorder characterized by recurrent arterial, venous and microvasculature thrombosis and/or obstetrical complications associated with circulating antiphospholipid antibodies (aPL) [1,2]. The diagnosis and classification of APS is based on the Sydney 2006 updated international classification criteria consensus [3]. According to these criteria, at least one of the clinical conditions and persistent detection of at least one of the criteria aPL have to be present for APS diagnosis [3] (Table 1). The autoantibodies accepted for the laboratory criteria include lupus anticoagulant, anticardiolipin and anti-β2-glycoprotein I IgG and/or IgM antibodies (Table 1). APS can be an isolated disease when it is defined as primary. Secondary APS represents coexistence of APS with some other autoimmune disorder, usually systemic lupus erythematosus (SLE) [1,4]. APS/SLE patients account for around 30% of all APS cases [5,6,7,8].

The estimated APS annual incidence and prevalence in the general population ranges between 1 and 2 cases per 100,000 persons and between 40 and 50 per 100,000 persons, respectively [9]. Most of the APS patients are diagnosed during the reproductive period with the mean age of diagnosis between 30 and 40 years for women, as several studies presented [6,8,9,10,11]. Moreover, APS is found to be more frequent in females especially when considering patients with secondary APS associated with SLE [6,9,10]. However, some studies found that there was no difference in APS frequency between sexes [7,8,9].

According to the clinical manifestations, two main subtypes of APS could be distinguished: vascular and obstetrical APS [12]. Vascular APS is mainly characterized by venous, arterial and small vessel thrombotic events in different organs [12]. Obstetrical APS (OAPS) is manifested with pregnancy morbidities and lower frequency of thrombotic events [12,13,14,15]. Distinct molecular signatures in these two APS subtypes were also found [16]. The most prevalent pregnancy complications in OAPS are early recurrent pregnancy loss (RPL), unexplained fetal death and stillbirth [5,14,17,18]. Complications in later stages of pregnancy including premature birth, preeclampsia (PE) and intrauterine growth restriction (IUGR) are also common for OAPS patients [5,14,17,18]. The original historic assumption was that complications in OAPS were associated with placental thrombotic phenomena [19]. However, experimental data accumulating over the past couple of decades have shown that inadequate placentation due to multiple detrimental effects of aPL on trophoblast, specialized placental cells, as well as other cell types of the placenta and uterus is a major cause of pregnancy morbidities in OAPS [12,14].

The gold standard treatment of APS is low dose aspirin combined with low molecular weight heparin at prophylactic or therapeutic doses, depending on a history of blood clots and previous complications during pregnancy [20,21]. In about 20–30% of OAPS patients, standard treatment does not give satisfactory results and they suffer from recurrent pregnancy complications [22]. There are several treatment options reserved for refractory OAPS including hydroxychloroquine, low-prednisone dose, intravenous immunoglobulins or plasma exchange [21]. Biologic therapies using anti-TNF-α antibodies in combination with standard treatment gave promising results for the treatment of refractory OAPS [22,23]. Recently, aPL-induced epigenetic modifications, including dysregulated expression of non-coding RNAs, emerged as key contributors to the APS progression as well as potential additional biomarkers and therapeutic targets in APS [24,25].

In this review, following the Introduction, we will briefly present general information on aPL types, their antigens and general mechanisms of action. Further, we will focus on aPL-induced effects on trophoblast cell survival and function. Finally, we will present current knowledge on non-coding RNAs as mediators of aPL-induced obstetric complications.

## 2. Antiphospholipid Antibodies

Antiphospholipid antibodies (aPL) are autoantibodies directed against phospholipids and/or phospholipid binding proteins present on cell membranes of various cell types, such as endothelial cells, leukocytes and platelets [26]. Laboratory criteria aPL, as mentioned above, include lupus anticoagulant, anticardiolipin and anti-β2-glycoprotein I (anti-β2GPI) antibodies [3]. In addition to these autoantibodies, aPL also include antibodies to annexin A5 [27], annexin A2 [28], protein S [29,30], phosphatidylethanolamine [31], lysobisphosphatidic acid [32], prothrombin [33] as well as autoantibodies to complexes, such as prothrombin/phosphatidylserine [34] and vimentin/cardiolipin [35], designated as non-criteria aPL. It was estimated that women with poor obstetric outcomes in 6-30% of all cases are carriers of aPL, either criteria and/or non-criteria [36,37,38,39].

A recent APS ACTION study has shown that a clinically meaningful aPL profile with positivity of all three laboratory criteria is associated with pronounced clinical features and more durable aPL for a period of 5 years in 78% of patients [40]. Within a spectrum of patients with clinical manifestations of APS, a significant portion was found not to meet these laboratory criteria. Patients without detectable criteria aPL are referred to as seronegative [41], while others that did not fulfill the aPL laboratory criteria regarding level or type of immunoglobulins are recognized as patients with non-criteria aPL, and/or lower level of criteria aPL [42].

Recently, regarding clinical obstetric manifestations, not much difference was noted in the cumulative incidence of adverse obstetrical events in seronegative and seropositive APS patients, although higher rates of intrauterine deaths (15% vs. 5%; *p* = 0.03), of PE (7% vs. 16%, *p* = 0.048) and lower live birth term (36 ± 3 vs. 38 ± 3 weeks of gestation; *p* = 0.04) were noted in seropositive APS patients [43]. The cumulative incidence of pregnancy complications was significantly decreased in treated versus untreated women with seronegative APS. A systematic review of studies comparing APS patients fulfilling Sydney criteria (definite APS) and non-criteria APS patients (NC-APS) presented that most studies have shown no significant difference in prevalence of clinical manifestations between definite and NC-APS patients including pregnancy morbidities [44]. A recent review focused on the evidence showing that non-criteria aPL may play a functional role in the signal transduction pathway(s) leading to thrombosis and pregnancy morbidity in seronegative APS patients [26]. The results of the recent retrospective multicenter study [45] from the European Registry on Obstetric Antiphospholipid Syndrome aimed at comparing clinical features, laboratory data and fetal–maternal outcomes between women with OAPS and with aPL-related obstetric complications not fulfilling Sydney criteria, yielded interesting conclusions pertinent to pregnancy outcomes in affected women that received treatment irrespective of fulfillment of aPL criteria. Treatment resulted in favorable obstetric outcomes for both the mother and the infant with no difference between OAPS and NC-OAPS. Given the shown obstetric benefits of treatment of patients with any aPL with clinical symptoms [43], it is worth stressing that OAPS, as the most frequent treatable autoimmune disease during pregnancy, should be treated irrespective of the fulfillment of the laboratory criteria.

Although accumulated data have shown a strong association of aPL and thrombosis, the underlying molecular mechanisms have not been completely elucidated and multiple mechanisms may be involved. It has been shown that aPL activates endothelial cells and promotes a proinflammatory and procoagulant cell phenotype through activation of Toll-like receptor 4/myeloid differentiation primary response 88 (TLR4/MyD88) signaling pathway, leading to the increased expression of adhesion molecules and release of cytokines [26,46]. Another mechanism of endothelial and monocyte cell activation by anti-β2GPI involves the upregulation of Tissue Factor which is a key molecule in extrinsic coagulation cascade initiation [47]. Platelets may also be activated by aPL, which leads to increased thromboxane A2 synthesis, glycoprotein IIb-IIIa expression and platelet factor-4 secretion [26]. It has also been demonstrated that annexin A5 protein functions as a physiological anticoagulant. It binds to phosphatidylserine on the cell surface forming a shield and prevents activation of procoagulant complexes [48].

In placental tissue, trophoblast cells abundantly express β2GPI [49], which together with hormonal and vascular changes linked to pregnancy highly contribute to the development of obstetrical complications in the presence of aPL. Besides trophoblast, decidual cells and other cell types at the feto–maternal interface highly express aPL antigens and can be affected by aPL as well. However, several lines of evidence described different roles of aPL in early and late pregnancy. Thus, in early pregnancy, aPL affect placentation and apoptosis of trophoblast cells, while thrombotic mechanisms are connected to late obstetric complications, such as IUGR and PE [1,50]. Moreover, it has been shown that anti-β2GPI binding to endothelial and trophoblast cells destroy the anticoagulant shield of annexin A5, thereby inducing a procoagulant state in placenta which can lead to thrombosis, thus influencing embryo fate [46,51].

At the level of trophoblast cells and the feto–maternal interface, various molecular mechanisms of aPL’s detrimental effects were proposed. Through the activation of TLRs and the NLR family pyrin domain containing 3 (NLRP3) inflammasome, aPL increase IL-1β and IL-8 trophoblast secretion [52,53]. It has been reported that aPL reduce beta-human chorionic gonadotropin (βhCG) production [54,55], signal transducer and activator of transcription 3 (STAT3) activity and interleukin (IL)-6 secretion, leading to decreased trophoblast function [56]. Several studies demonstrated a complement activation by aPL, which leads to the release of reactive oxygen species, antiangiogenic factors, Tissue Factor as well as TNF-α [57,58,59]. Due to the action of aPL, placentas of APS patients are structurally modified as a consequence of aPL internalization by syncytiotrophoblast [60], which will be discussed in the next section.

Clearly, there are many different mechanisms involved in APS during pregnancy. Therefore, the knowledge about different molecular mechanisms triggered by aPL involved in the abnormal development of placenta and placental dysfunction was and will be mostly useful in terms of OAPS management.

## 3. The Impact of aPL on Trophoblast Cells

**Figure 1:**
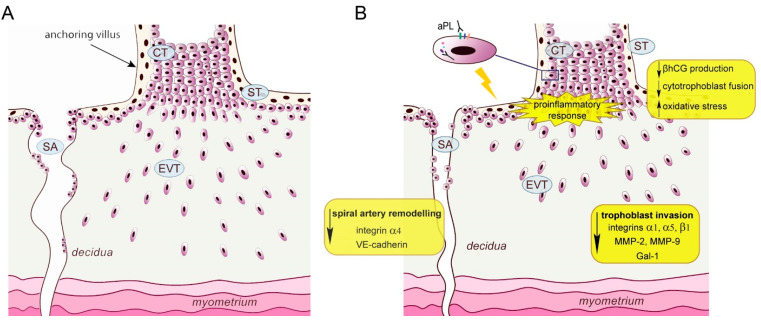
(**A**). Schematic presentation of the anchoring chorionic villus and trophoblast invasion into the decidual tissue in healthy pregnancy. (**B**). Possible mechanisms of antiphospholipid antibodies’ action on trophoblast cells leading to defective placentation/placental malfunction. aPL—antiphospholipid antibodies; CT—cytotrophoblast; ST—syncytiotrophoblast; EVT—extravillous trophoblast; SA—spiral artery; MMP-matrix metalloproteinase; Gal-1—galectin-1; βhCG—β-human chorionic gonadotropin.

Placenta is a unique organ essential for pregnancy success, which forms contact between the mother and fetus and exerts multiple important functions. Aberrant placentation is associated with diverse pregnancy complications such as miscarriage, stillbirth, pre-term labor, IUGR and PE [61]. Trophoblast cells are specific placental cells, exerting a variety of functions at the feto–maternal interface. Placental chorionic villi are covered with syncytiotrophoblast, a multinucleated trophoblast layer directly facing maternal circulation, which facilitates nutrient transport and gas exchange between mother and fetus. Syncytiotrophoblast secretes hormones necessary for the maintenance of healthy pregnancy such as βhCG and placental lactogen [61]. Underneath syncytium, there is a layer of cytotrophoblast cells which continuously proliferate and through constant fusion form syncytiotrophoblast. Another type of trophoblast cells are extravillous trophoblast cells (EVTs), which also differentiate from cytotrophoblast through the process of epithelial-mesenchymal transition (EMT). Detaching from the tips of the anchoring villi, EVTs invade the maternal decidual stroma, spiral arteries and other luminal structures in the uterus, which is essential for the process of placentation [61,62] (Figure 1A). The outcome of this process is adequately attached placenta and modified spiral arteries that enable sufficient supply of oxygen, nutrients and other factors to the developing fetus.

Trophoblast cells abundantly express aPL antigens, especially β2GPI [49]. Anti-β2GPI antibodies are considered to be the main contributors to the pathogenesis of OAPS and a great number of in vitro studies deciphering pathogenic mechanisms of the syndrome were conducted using these autoantibodies. It was found that aPL induce detrimental effects on trophoblast cells affecting their proliferation, differentiation and survival [63,64], as well as other cellular processes including invasion and migration [65,66,67,68,69] through different molecular mechanisms which will be discussed in this section.

### 3.1. Trophoblast Survival

Apoptosis has an important role in normal placental development [70,71]. However, increased rates of trophoblast apoptosis are associated with placental diseases [70,71]. In the past two decades, a growing body of evidence suggests that aPL affect trophoblast cell proliferation and apoptosis. In vitro studies on rat’s embryos demonstrated that purified aPL IgG treatment inhibited embryo and yolk sac growth and increased apoptosis of ectoplacental cone giant cells [72,73]. In mice, passive immunization with human aPL during gestation attenuated placental morphogenesis [69,74], reduced trophoblast proliferation [74] and increased placental apoptosis as evidenced by the increased index in TUNEL-positive cells and pronounced DNA-fragmentation [75].

Analyzing sections of human first trimester placentas after elective abortions and aPL-associated miscarriages, Bose et al. found that aPL-associated trophoblast development is aberrant [76]. Authors hypothesized that aPL may stimulate the premature onset of cytotrophoblast proliferation and differentiation, in favor of syncytial fusion, which could result in accelerated exhaustion of cytotrophoblast ‘stem’ cells, leading to the altered morphology and function of placenta and consequential pregnancy loss [76]. Furthermore, in vitro experiments using human placental explants from the first trimester of pregnancy cultured with sera of APS/SLE patients with history of RPL showed reduced placental villi growth and trophoblast cell proliferation as well as increased trophoblast apoptosis in treated explants [73,77]. During pregnancy, as part of normal placental aging, the syncytiotrophoblast layer sheds through the process of apoptosis releasing multinucleated syncytial aggregates known as trophoblast debris in maternal circulation [78]. Studies on the human placental explant model showed that aPL treatment increased trophoblast shedding rates and altered the cell death process through which trophoblast debris is formed [60,79]. It has been shown that aPL antibodies affected mitochondrial function through mitochondrial leakage and cytochrome C release, which eventually led to necrotic cell death and extrusion of necrotic trophoblast debris [60]. After phagocytosis, aPL-induced trophoblast debris stimulated activation of endothelial cells which could explain, at least in part, how aPL could increase the risk of PE and other adverse pregnancy outcomes associated with an activated endothelium in OAPS patients [60,79]. An additional proposed mechanism by which aPL could cause cell death in the syncytiotrophoblast is the interruption of placental lipid signaling and decreased expression of protein kinase C-epsilon (PRKCE) as determined on the placental explant model [80].

Moreover, aPL-induced transcriptome and metabolome alterations connected to trophoblast cell death have been found [80,81]. Transcriptomic analysis revealed changes in the expression of factors involved in the regulation of apoptosis, including *BCL2L1*, *MCL1*, *PDCD2L*, *FASLG*, *SEMA6A*, *PRKCE* and *TRAIL* mRNAs in response to aPL treatment of human placental explants [81]. Altered lipid metabolism, especially of ceramides and diacylglycerols, important players in cell death regulatory pathways, was the most pronounced aPL-induced metabolic change detected in treated human placental explants [80]. Expression of anti-apoptotic *BCL2* and pro-apoptotic *BAX* genes on mRNA and protein levels was altered in primary trophoblast cells after aPL treatment [82]. Reduced BCL2/BAX ratio indicating a pro-apoptotic state was detected but with no change in apoptosis rates as evidenced by DNA fragmentation or positivity for the caspase-cleaved epitope of cytokeratin-18 cytoskeletal protein (M30) [82]. In agreement with that, results from our laboratory showed that aPL moderately increased proliferation of the EVT cell line HTR-8/SVneo with no change in the rate of apoptosis [83]. On the other hand, Mulla and colleagues showed that lower concentrations of aPL also moderately stimulated proliferation of HTR-8/SVneo cells but as aPL concentration increased, viability of trophoblast cells were significantly reduced mediated by increased activity of caspase-8, caspase-9 and caspase-3 [52]. An aPL-induced HTR-8/SVneo cell death was at least partially due to increased proinflammatory response of HTR-8/SVneo cells to aPL treatment [52]. Discrepancy between results of different studies on the extent of aPL-induced effects on trophoblast cell survival are most probably due to the heterogeneity of aPL used in experiments as well as the duration of treatment.

Taken together, these observations suggest that apoptosis might be an important mechanism in aPL-induced defective placentation in OAPS, without necessarily involving thrombotic phenomena. 

### 3.2. Trophoblast Cell Function

Along its invasive pathway, trophoblast undergoing EMT acquire markers of an invasive phenotype—integrins α5β1 and α1β1 (forming fibronectin and laminin/collagen receptors), among others, and start to secrete proteolytic enzymes, of which matrix metalloproteinases (MMP) -2 and -9 have the most important role [84,85]. A subpopulation of EVTs that invades the spiral arteries, designated endovascular trophoblast, has been shown to express specific endothelial markers, such as integrin αVβ3, VE-cadherin and α4 integrins [85,86]. 

A number of in vitro studies on trophoblast cells showed that aPL directly alter trophoblast cell function including invasion and migration abilities. IgG isolated from patients with APS as well as monoclonal antibodies reactive with β2GPI suppressed trophoblast invasion, as shown using several trophoblast cell models including primary trophoblast isolated from first or third trimester placenta, as well as normal trophoblast HTR-8/SVneo and choriocarcinoma JAR cell lines [65,66,67,68,69]. Moreover, Poulton et al. showed that IgG purified from OAPS patients but not from vascular APS patients inhibited HTR-8/SVneo invasion expressing the difference between these two APS subtypes [87].

Further investigations showed that aPL-associated reduction in invasiveness could at least, in part, be attributed to downregulation in invasion mediators—integrin subunits α1, α5 and β1 [67,68,88,89]. MMP-2 and MMP-9 are highly expressed during implantation and early stages of pregnancy, playing a key role in the degradation of the extracellular matrix (ECM) by trophoblast cells [90,91]. Anti-β2GPI antibodies inhibited MMP-2 and MMP-9 secretion by trophoblast cells [66,68], while IgG from aPL-positive sera (aPL IgG) significantly decreased the level of MMP-9 and the overall gelatinolytic capacity of HTR-8/SVneo cells as assessed by in situ gelatin zymography [89]. Another possible mechanism of aPL-mediated invasion suppression is through downregulation of IL-6 secretion and STAT3 activity, as this was shown for mouse anti-β2GPI antibodies in HTR-8/SVneo cells [56]. This cytokine acts as an important regulator of the implantation and placentation processes [56]. Both mouse anti-β2GPI and patient-derived aPL induced an inflammatory response in trophoblast cells, which may result in a negative impact on trophoblast cell function, including an invasive capacity [52,92,93,94].

Our previous findings further support the hypothesis of the direct negative effect of aPL on trophoblast invasion process. Galectin-1 (Gal-1), lectin abundantly present at the feto–maternal interface, is an important part of the trophoblast invasion machinery, as it modulates trophoblast adhesive and invasive capacities [95]. This lectin has the ability to bind various ECM proteins, as well as cell surface adhesion molecules, including trophoblast integrin β1 [96]. Results obtained in our previous research on HTR-8/SVneo cells showed that Gal-1 was reduced in conditioned media of aPL IgG-treated cells, while total cell protein remained unaltered, suggesting that aPL IgG may affect Gal-1 secretion in a manner not yet elucidated [89]. We have shown that aPL IgG treatment activates the p38 MAPK signaling pathway, and that inhibitory effects on integrin subunits and secreted Gal-1 were dependent on this activation [89]. The role of the p38 MAPK signaling pathway was shown in multiple processes involving trophoblast, such as the stimulation of trophoblast cell motility by EGF, demonstrated in experiments on EVT cell line SGHPL-4 [97].

Furthermore, aPL seem to interfere with the trophoblast ability to differentiate into an endothelial-like phenotype contributing to aPL-associated aberrant spiral artery remodeling. Placentas of mice treated with anti-β2GPI antibodies during gestation showed a number of pathological changes including defective vascular remodeling [69]. Moreover, in vitro experiments detected that anti-β2GPI antibodies inhibited the HTR-8/SVneo tube formation ability [69] and compromised the trophoblast ability to bind and integrate into the endothelium [98]. Previous research showed that treatment of primary term trophoblast with aPL IgG downregulated VE-cadherin expression [88], an adhesion molecule important for trophoblast–endothelial interaction, endovascular invasion and spiral artery remodeling [99,100]. Moreover, integrin subunit α4 was also decreased by the treatment [89]. This integrin subunit is upregulated in endovascular trophoblast, probably as one of the adhesion molecules needed to facilitate adhesion to vascular epithelia, where α4β1 integrin is proposed to bind the vascular cell adhesion molecule-1 (VCAM-1) [86]. The schematic representation of the possible mechanisms of aPL action at the site of implantation, based on in vitro findings is given in Figure 1B.

Recently, Yes-associated protein (YAP), the transcription co-activator of the Hippo signaling pathway, was proposed to act as key effector molecule which links aPL-induced upstream intracellular signals and alteration of different trophoblast functions [101]. Namely, it was detected that aPL treatment decreased YAP protein levels in HTR-8/SVneo cells [101]. YAP downregulation increased apoptosis, inhibited migration, invasion and tube formation ability of HTR-8/SVneo cells [101,102,103]. Previous studies of early implantation and trophoblast development showed that inhibition of YAP reduced endometrial attachment, outgrowth, and trophoblast gene expressions of human embryonic stem cell–derived trophoblastic spheroids [104]. Furthermore, decreased YAP expression levels were found in PE placentas [102,103]. All the data present the importance of YAP regulation of trophoblast function, indicating the diversity of effects aPL-induced YAP downregulation could have in establishment and maintenance of pregnancy.

In conclusion, the findings of our and other groups’ research suggest that aPL may induce defective placentation by reducing trophoblast invasion through inhibiting effector molecules—integrins and MMPs and by limiting the amount of Gal-1 present extracellularly. Furthermore, these autoantibodies may interfere with the modification of spiral arteries through downregulation of adhesion molecules characteristic for endovascular trophoblast. Other mechanisms are not excluded.

### 3.3. Internalization of aPL in Trophoblast Cells

In addition to cell invasion, other cell functions specific for trophoblast have also been shown to be negatively influenced by aPL. Cytotrophoblast fusion and production of a major trophoblast-derived hormone βhCG, necessary for pregnancy progression, were suppressed by aPL [54,105,106,107]. Additionally, a recent study linked aPL to syncitiotrophoblast oxidative stress [108]. This is in accordance with the high heterogeneity of these antibodies. What is still not well understood is the exact mechanism through which these autoantibodies initiate these cellular responses, but both cell surface and intracellular antigens have been proposed as targets.

The ability of patient-derived autoantibodies to penetrate living cells was first proposed more than 40 years ago [109]. Since then, this property was shown for a number of autoantibodies, which led to the conclusion that intracellular antigens are not immunologically privileged, as first thought to be. Several studies have demonstrated that different types of aPL may be detected intracellularly. A study by Galve-de Rochemonteix and colleagues (2000) showed that aPL are accumulated in late endosomes of baby hamster kidney (BHK) cells in culture [110]. A more recent study demonstrated that anti-phosphatidylethanolamine antibodies target the cytosolic surface of early endosomes in human umbilical vein endothelial cells (HUVECs) and hypothesizes that these antibodies could have important implications for a wide range of biological processes in different cell types [111]. Trying to elucidate the mechanism of interaction between anti–β2GPI monoclonal antibodies WB-6 with resting monocytes, Virachith and colleagues (2019) found that WB-6 exhibits binding activity to DNA and enters living monocytes [112]. 

It is well known that the passive immunity provided to the human fetus is in part obtained through the transport of maternal IgG across the syncytiotrophoblast. Even though the transport of maternal IgG increases after the 22nd week of gestation and is mediated by the Fcγ receptor, there are data indicating that transport across early placental trophoblast is not limited by a lack of specific IgG receptors. Furthermore, aPL have been shown to bind EVTs [68,113,114], and also to bind and internalize into syncytiotrophoblast [60]. This process was independent on Fc-receptor, as shown by employment of anti-β2GPI that lacks Fc fragment but was dependent on the low-density lipoprotein receptor (LDLR) [60]. Extrusion of necrotic trophoblast debris from syncytiotrophoblast, caused by aPL treatment, was dependent on the internalization of these antibodies [60]. A subsequent study from the same group showed that the same aPL are not internalized in the EVTs from explanted villi [114]. Our study, however, demonstrated that patient-derived aPL IgG was able to enter and accumulate in the primary first trimester cytotrophoblast in culture and HTR-8/SVneo cells in a time-dependent manner [113], similarly to findings of Hou and colleagues on HUVECs [111]. We suspect that this discrepancy could be due to the longer exposure time in our approach and, to a lesser degree, to the difference in species of origin and specificity of the used antibodies.

Based on the collected data from studies on the aPL potential to enter trophoblast as well as other types of cells, it can be concluded that there is a whole spectra of possible cell surface as well as intracellular targets for these autoantibodies, implicating other possible routes for aPL’s influence on trophoblast cell function.

## 4. Non-Coding RNAs—Emerging Players in OAPS Pathophysiology

Being multifunctional regulators of different biological processes, as the growing body of evidence suggests, it is not surprising that there is increasing interest in studying the involvement of non-coding RNAs (ncRNAs) in the pathophysiology of APS and other autoimmune disorders [25,115,116,117,118,119,120,121]. NcRNAs, including microRNAs (miRNAs) and long non-coding RNAs (lncRNAs), regulate gene expression on transcriptional and post-transcriptional levels [122,123]. Aberrant expression of ncRNAs could significantly affect various cellular processes eventually leading to the development of different pathologies [124,125,126].

An in vitro study on trophoblast HTR-8/SVneo cells revealed that anti-β2GPI upregulated cellular and exosome levels of miR-146a-5p, miR-146a-3p, miR-155 and miR-210 [93]. Moreover, women positive for aPL experiencing pregnancy complications had elevated circulating miR-146a-3p levels compared to healthy controls [93]. Previous research showed that aPL stimulated a proinflammatory response in trophoblast cells [52]. Specifically, anti-β2GPI elicited IL-8 secretion among other cytokines in HTR-8/SVneo cells through the TLR4/MyD88 pathway [52]. Since miR-146a-5p, miR-155 and miR-210 were shown to mediate TLR signaling [127,128,129,130], Gysler and colleagues investigated the role of these miRNAs in TLR4-dependent IL-8 secretion in aPL-treated trophoblast cells [93]. They showed that aPL-stimulated upregulation of miR-146a-5p, miR-146a-3p and miR-210 but not miR-155 was TLR4 dependent. However, anti-β2GPI-induced IL-8 secretion was shown to be mediated by miR-146a-3p and it was through the activation of RNA sensor TLR8 [93]. 

MiR-146a, miR-155 and miR-210 are important regulators of numerous cellular processes. Although there are some studies with opposing results [131,132], a number of studies showed that overexpression of miR-146a [133,134,135,136], miR-155 [137,138,139] and miR-210 [140,141,142,143] inhibited migration and invasion of trophoblast cells. MiR-146a and miR-155 are major regulators of the immune response and disrupted expression of these miRNAs has been associated with pathologies characterized by chronic inflammation [144]. MiR-146a affects trophoblast EMT, migration and invasion abilities through direct downregulation of TNF receptor-associated factor 6 (TRAF6) [134] and atypical chemokine receptor 2 (ACKR2) [136]. TRAF6 is a signal transducer in the TLR4/MyD88 pathway involved in the regulation of the immune response [145,146] but also implicated in different cellular processes including regulation of proliferation, migration and invasion of cancer cells [146]. ACKR2, a chemokine scavenger, is involved in the maintenance of balance between pro and anti-inflammatory cytokines at the feto–maternal interface [147] and dysregulation of this molecule is associated with different pregnancy complications [148,149]. Wnt/β-catenin signaling pathway was also found to be affected by miR-146a-5p through direct regulation of Wnt2 expression [135]. Furthermore, it was shown that overexpression of miR-146a downregulated CXCR4 and EGFR in HTR-8/SVneo cells [133] affecting signaling pathways activated through these two receptors which are involved in regulation of trophoblast migration and invasion [150,151]. MiR-155 affects the TGF-β/Smad signaling pathway important for regulation of EVT invasion [152] directly targeting expression of Smad2 [153]. Other direct miR-155 targets were also implicated in the regulation of trophoblast cell functions including angiogenic factor CYR61 [137], an important regulator of cell cycle progression cyclin D1 [154], eNOS [139] and forkhead-box class O transcription factor 3 (FOXO3) [155].

MiR-210 is a master hypoxamiR, a miRNA whose expression is induced by hypoxic conditions [156]. It is an important regulator of mitochondrial metabolism, cell proliferation and differentiation, angiogenesis and other oxygen-sensitive processes [156]. Overexpression of miR-210 in trophoblast cells inhibited mitochondrial respiration which consequently could lead to generation of excessive amounts of reactive oxygen species and increased placental oxidative stress [157]. MiR-210 inhibited HTR-8/SVneo invasion via ERK/MAPK-dependent mechanism [140]. Overexpression of miR-210 dysregulated expression of EMT-related proteins and consequentially inhibited invasive abilities of trophoblast cells [143]. Namely, expression of the mesenchymal marker vimentin and N-cadherin, a promoter of EMT, was decreased while E-cadherin, an epithelial marker, was upregulated in HTR-8/SVneo cells overexpressing miR-210 [143]. Moreover, trophoblast upregulation of miR-210-3p has been associated with impaired remodeling of spiral arteries [142]. In this study, authors showed that miR-210-3p-dependent impairment of trophoblast function is mediated through direct dysregulation of caudal-related homeobox transcription factor 2 (CDX2), essential transcription factor for trophoblast differentiation active during blastocyst development [142].

Reduced trophoblast invasion and spiral artery remodeling are associated with pregnancy complications characteristic for OAPS patients, such as RPL and PE [14,17]. Since increased levels of miR-146a, miR-155 and miR-210 have been found in placentas of aPL-negative RPL [134,158,159,160] and PE patients [135,136,137,139,161,162,163] it could be concluded that aPL-induced upregulation of named miRNAs in trophoblast cells is a contributing factor to the development of these obstetric complications in OAPS patients. Moreover, increased levels of miR-146a, miR-155 and miR-210 were found in blood of patients suffering from pregnancy loss and/or PE [140,164,165,166,167] suggesting the use of these miRNAs as biomarkers for early diagnostics as well as mechanism-based targets of new therapeutics for RPL and PE associated or not with OAPS.

As mentioned above, APS is one of the main risk factors for RPL [168] but underlying mechanisms of aPL-induced RPL are still not completely elucidated. Recent studies identified lncRNA MALAT1 as one of the major regulators of the processes important for the adequate placental development and function in early pregnancy [158,169,170,171,172,173]. Namely, MALAT1 levels were significantly downregulated in placentas of the patients experiencing RPL of unknown etiology compared to the healthy controls [158,174]. Furthermore, recent research showed that trophoblast and placental MALAT1 levels of aPL-positive RPL patients were even lower than MALAT1 levels of aPL-negative RPL patients [172]. Furthermore, aPL-positive RPL mouse model was generated [172]. The embryo resorption rate was increased in aPL-positive RPL mice compared both to RPL mice negative for aPL and control mice [172]. Placental MALAT1 overexpression by adenoviral transfection in aPL-positive RPL mice significantly decreased embryo resorption rate compared to untreated aPL-RPL mice [172].

Moreover, low expression levels of MALAT1 were also found in placentas of PE patients comparing with normal controls [170,171,173,175]. Effects of decreased MALAT1 expression on trophoblast cell function were investigated in vitro by downregulation of this lncRNA in HTR-8/SVneo and JAR cells. The results showed that trophoblast cells with decreased MALAT1 levels proliferated less than unmodified cells and their migrating and invasive abilities were decreased as well as expression of EMT-related proteins [158,169,170,171,172,173]. Different mechanisms of action were proposed for MALAT1-dependent regulation of trophoblast cell function. It was shown that MALAT1 modulates IGF-1/PI3K/Akt signaling affecting trophoblast migration and invasion abilities [169]. Furthermore, VEGFA was found to be a downstream mediator of MALAT1-depepndent inhibition of trophoblast proliferation [173] and endovascular differentiation [171].

Among other mechanisms of action, lncRNAs can regulate gene expression by affecting miRNA expression and activity via sequestration [123,176]. Some lncRNAs, part of competing endogenous RNA (ceRNA) family, act as molecular miRNA sponges. They competitively bind specific miRNAs and thus prevent them from binding to their target mRNAs. In that way, negative miRNA effect on target gene expression is reduced [123,176]. Mutual regulation of miRNA and lncRNA activities is involved in regulation and fine tuning of many biological processes. Growing body of evidence has shown that MALAT1 functions as ceRNA [158,169,173,177,178,179,180,181,182]. Among other miRNAs, miR-146a [158,177,178,179] and miR-155 [183,184] have also been shown to be MALAT1 binding partners. These studies indicate the involvement of MALAT1/miR-146a and/or miR-155 regulatory axes in diverse negative effects of aPL on placental function leading to RPL or other obstetrical complications in OAPS.

Recently, another lncRNA named LncNR_040117 has been identified as an important mediator of APS-induced RPL [24]. Firstly, it has been shown that platelet-derived microparticles (PMPs) isolated during first trimester of pregnancy from APS patients with the history of RPL stimulated apoptosis and inhibited invasion and migration of trophoblast HTR-8/SVneo cells [185]. PMPs are vesicles derived from platelets undergone activation or apoptosis [186]. They are the most abundant type of microparticles present in human circulation and they were associated with different pathologies such as cancer, cardiovascular and autoimmune diseases [186,187,188]. PMPs mediate various physiological processes affecting target cells thorough specific interactions including surface receptor signaling and delivering of bioactive molecules such as cytokines, enzymes, growth factors and RNAs [186,187,188,189,190]. Content of PMPs’ cargo molecules depend on the signals activating platelets and stimulating PMPs’ formation and it is modified in pathological conditions [186,187,188]. lncRNA profiling of APS-associated PMPs identified LncNR_040117 as one of the significantly overexpressed lncRNAs in PMPs isolated from APS patients with the history of RPL comparing to the gestational age matched healthy controls [24]. Moreover, this lncRNA has been proposed for biomarker of APS-induced RPL [24]. Downregulation of LncNR_040117 stimulated proliferation, migration and invasion of transfected HTR-8/SVneo cells [24,191]. These results indicate that LncNR_040117 upregulation by intake of APS-related PMPs could have opposite, detrimental effects on trophoblast function. On the other hand, finding strategies for targeted blocking of this lncRNA could be a potential method of preventing miscarriage in APS patients. The possible implications of APS-associated ncRNAs in trophoblast cell function are summarized in Table 2.

NcRNA research field is constantly developing, giving insights in complex regulation of biological processes in health and disease. Future elucidation of various participants in these fine-tuned processes will provide new opportunities for the development of potential therapeutics and strategies for management of obstetrical complications related to APS.

## 5. Conclusions

Given that APS still represents one of the most common threats for pregnancy complications, the current knowledge regarding placental dysfunction in APS must be significantly improved. There are multiple possible mechanisms involved in APS-associated placental dysfunction. According to the evidence from in vitro and in vivo studies, both extracellular and intracellular antigens may be targeted by aPL, activating different cellular responses that further cause excessive apoptosis and impaired trophoblast invasion/placentation. The aPL-induced modulation of epigenetic mechanisms such as changing ncRNAs expression is emerging as a key contributor to APS progression. 

Current treatment strategies are not effective for all patients. Conventional treatment strategies mostly include antithrombotic agents, while immunosuppressive therapy has been increasingly used. Non-coding RNAs are emerging players in the pathogenesis of APS. Given that these molecules have been proposed as biomarkers of many pathological conditions and as therapeutic targets, future investigations could be directed towards identification of differentially expressed ncRNAs in APS, and elucidation of their roles in the pathogenesis of APS. This would provide basis for the development of ncRNA-targeting treatments. Several ncRNA-targeting drugs for other conditions are already being tested in clinical trials.

Taken together, further investigations are needed to fully understand the causes of APS-associated pregnancy complications, so that every affected woman can be treated adequately.

## Figures and Tables

**Table 1:** Criteria for diagnosis of APS.

APS Disease Classification Criteria According to Sydney Protocol	
Clinical Criteria (at Least 1 of 2)	Laboratory Criteria (at Least 1 of 3)
Vascular thrombosis: ≥1 clinical episode of thrombosis in any tissue/organ, arterial or venous Pregnancy morbidity: ≥1 morphologically normal fetal loss, ≥10th week of gestation, or ≥1 premature birth of a normal neonate before the 34th week due to (i) eclampsia or severe preeclampsia or (ii) placental insufficiency, or ≥3 unexplained consecutive spontaneous abortions < 10th week of gestation (with exclusion of parental anatomic, hormonal or chromosomal causes).	Presence of (at least twice in min. 12 weeks): Lupus coagulant (LA) Antibody to cardiolipin (aCL), β-2 glycoprotein I (anti-β2GPI) (high titer, IgG or IgM) Classification based on laboratory tests: Type I: >1 laboratory criterion present (any combination) Type IIa: LA antibodies only Type IIb: aCL antibodies only Type IIc: anti-β2GPI antibody only

**Table 2:** Possible implication of APS-associated ncRNAs in trophoblast cell function.

ncRNA	APS-Associated ncRNA Dysregulation	Implication in Trophoblast Function
miR-146a-5p miR-146a-3p miR-155 miR-210	Upregulated in anti-β2GPI treated HTR-8/SVneo cells [93]	Overexpression of miR-146a [133,134,135,136], miR-155 [137,138,139] and miR-210 [140,141,142,143] inhibited invasion and migration of HTR-8/SVneo cells
		Overexpression of miR-210 inhibited mitochondrial respiration in primary EVT cells [157]
		Overexpression of miR-210-3pinhibited tube formation of HTR-8/SVneo cells [142]
lncRNA MALAT1	Downregulated in placentas of APS-induced RPL patients [172]	MALAT1 downregulation inhibited proliferation, migration and invasion of HTR-8/SVneo cells [158,169,170,171,172]
LncNR_040117	Upregulated in PMPs isolated from APS-induced RPL patients [24]	LncNR_040117 downregulation stimulated proliferation, migration and invasion of HTR-8/SVneo cells [185,191]

EVT—extravillous trophoblast; PMPs—platelet-derived microparticles; RPL—recurrent pregnancy loss.

## Data Availability

No new data were created or analyzed in this study. Data sharing is not applicable to this article.
